# Gastric Ischemia Secondary to Atherosclerotic Disease: A Case Report and Literature Review

**DOI:** 10.7759/cureus.116256

**Published:** 2026-09-15

**Authors:** Mohamad El Haress, Hind Joumaa, Hana Diab, Alaa Hajjar, Fatima Alsalman, Walid Nasreddine

**Affiliations:** 1 Department of Surgery, American University of Beirut Medical Center, Beirut, LBN; 2 Gastroenterology and Hepatology Department, Makassed General Hospital, Beirut Arab University, Beirut, LBN; 3 Gastroenterology Department, Makassed General Hospital, Beirut Arab University, Beirut, LBN

**Keywords:** case report, chronic mesenteric ischemia, endovascular revascularization, gastric ischemia, ischemic gastritis, superior mesenteric artery

## Abstract

Gastric ischemia is an uncommon entity, as the stomach has an extensive collateral arterial blood supply; clinically significant injury usually requires multivessel mesenteric compromise. When it occurs, it closely mimics peptic ulcer disease and gastric malignancy, and diagnosis is often delayed. A 77-year-old female patient with poorly controlled type 2 diabetes mellitus and dyslipidemia (non-compliant with metformin and atorvastatin) presented with a one-month history of severe postprandial epigastric pain, progressive food avoidance, and 10 kg of unintentional weight loss. She was admitted for pain management and further evaluation. Upper GI endoscopy revealed severe erosive gastroduodenitis with multiple gastric ulcers and a large duodenal ulcer. Tests for *Helicobacter pylori* were negative. Nonsteroidal anti-inflammatory drug use was not reported. Histopathology was negative for malignancy. Serum lactate and lactate dehydrogenase were within normal limits. Contrast-enhanced computed tomography angiography showed severe multivessel atherosclerotic disease, with near-complete occlusion of the coeliac trunk, a 3-cm segment of severe superior mesenteric artery stenosis with areas of near-complete luminal occlusion, and 40% stenosis of the inferior mesenteric artery. At multidisciplinary review, the diagnosis of chronic mesenteric ischemia presenting as ischemic gastroduodenitis was made, and the patient underwent endovascular angioplasty with stenting of the superior mesenteric artery with resolution of symptoms and complete endoscopic mucosal healing. She was discharged on dual antiplatelet therapy (acetylsalicylic acid and clopidogrel) in addition to fenofibrate, atorvastatin, and strict low-fat diet. At six-month follow-up, she was asymptomatic with a normal complete blood count and liver enzyme panel, and her lipid profile had markedly improved from admission. Chronic ischemic gastroduodenitis should be considered in elderly patients with postprandial pain, weight loss, and diffuse *H. pylori*-negative gastroduodenal ulceration. Normal serum lactate does not exclude the diagnosis. Revascularization of the isolated superior mesenteric artery can reverse ischemic gastric injury even with coeliac artery occlusion.

## Introduction

Gastric ischemia is a rare condition that can be life-threatening due to the extensive collateral arterial supply to the stomach from the coeliac axis via the left and right gastric, gastroepiploic, and short gastric arteries. Therefore, clinically significant ischemic injury is usually only seen with severe vascular compromise, systemic hypoperfusion, or multivessel mesenteric arterial disease [1,2]. The most common cause of chronic mesenteric ischemia (CMI) is progressive atherosclerotic stenosis or occlusion of the coeliac artery and/or superior mesenteric artery (SMA). Mesenteric artery occlusive disease is relatively common in elderly patients with cardiovascular risk factors, but symptomatic CMI is uncommon due to extensive collateral circulation. The classic clinical presentation includes postprandial abdominal pain, avoidance of food, and progressive weight loss, often resulting in delayed diagnosis due to nonspecific symptoms [2-4]. Gastric ischemia is a rare presentation of CMI and often mimics more common gastrointestinal disorders such as peptic ulcer disease, erosive gastritis, and upper gastrointestinal malignancy. The endoscopic appearance is variable and may reveal diffuse erythema, mucosal oedema, erosions, deep ulceration or, in advanced cases, transmural necrosis and perforation. Histopathology is mainly useful to exclude malignancy, infectious causes, and drug-induced injury rather than to establish the diagnosis of ischemia [1,5]. In suspected mesenteric ischemia, computed tomography angiography (CTA) has become the imaging modality of choice as it detects reliably arterial stenosis or occlusion and, at the same time, provides information on bowel and gastric perfusion. Current guidelines in vascular surgery recommend endovascular revascularization as the preferred initial approach for the majority of patients with atherosclerotic CMI due to its association with reduced peri-procedural morbidity, shorter hospitalization, and excellent technical success; however, long-term surveillance for restenosis is still required [2,4,6]. We report a case of chronic ischemic gastroduodenitis with severe ulceration of the stomach and duodenum secondary to severe stenosis of the SMA and near-complete occlusion of the coeliac artery. Endoscopic healing and complete clinical recovery followed successful endovascular revascularization. This case emphasizes the need to consider gastric ischemia in the differential diagnosis of elderly patients with refractory upper gastrointestinal ulceration, weight loss, and postprandial abdominal pain.

## Case presentation

A 77-year-old woman was referred for elective upper gastrointestinal endoscopy with a one-month history of progressive postprandial epigastric pain. The pain was severe, beginning 15 to 30 minutes after eating and lasting one to two hours before resolving spontaneously. It became progressively disabling, and the patient gradually reduced her oral intake for fear of provoking symptoms. In the week prior to presentation, she was only able to tolerate a full liquid diet. During this time, she lost about 10 kg unintentionally. She denied nausea, vomiting, hematemesis, melena, change in bowel habits or any other associated gastrointestinal symptoms. Past medical history was significant for type 2 diabetes and dyslipidemia. She had been on metformin 1,000 mg once daily for two years and atorvastatin 40 mg once daily for 20 years but admitted to longstanding non-adherence to both medications. She had a history of smoking 60 pack-years (2 packs/day for 30 years) and was an active smoker at the time of presentation. She had no history of hypertension, coronary artery disease, or peripheral artery disease. She denied taking nonsteroidal anti-inflammatory drugs, aspirin, corticosteroids, or anticoagulants, and denied alcohol use. She had no prior history of peptic ulcer disease or *Helicobacter pylori* infection, no family history of gastric cancer, diabetes, or dyslipidemia. Laboratory evaluation one year prior to presentation showed a persistently atherogenic lipid profile: total cholesterol 268 mg/dL (desirable <200 mg/dL), low-density lipoprotein (LDL) cholesterol 179 mg/dL (optimal <100 mg/dL), high-density lipoprotein (HDL) cholesterol 38 mg/dL (desirable >50 mg/dL in women), non-HDL cholesterol 230 mg/dL (desirable <130 mg/dL), and triglycerides 247 mg/dL (normal <150 mg/dL). Glycated hemoglobin (HbA1c) was 7.2% (normal <5.7%) at that time. On examination, the patient was emaciated. Vital signs were blood pressure 106/64 mm Hg, pulse 98/min regular, respirations 18/min, temperature 36.6°C, and oxygen saturation 98% on room air. She was 160 cm tall and weighed 47.5 kg, with a body mass index of 18.6 kg/m^2^ (underweight). She had an unintentional weight loss of 10 kg and weighed approximately 57.5 kg (body mass index of 22.5 kg/m^2^, within the normal range) about one month before the onset of symptoms. There was mild epigastric tenderness on deep palpation but no guarding or rebound tenderness or palpable mass. The abdomen was soft and non-distended. The pain reported was grossly out of proportion to the examination findings. Bowel sounds were normal, and no epigastric bruit was heard. Peripheral pulses were normal, and no clinical evidence of peripheral vascular disease was present. Rectal examination was normal, and fecal occult blood test was negative; initial lab workup was within normal limits with normal liver enzymes, normal pancreatic enzymes, and complete blood count. Alanine aminotransferase was 22 U/L (7-35 U/L), aspartate aminotransferase was 25 U/L (8-33 U/L), gamma-glutamyl transferase was 28 U/L (9-36 U/L) and alkaline phosphatase was 76 U/L (35-104 U/L). Total bilirubin was 0.7 mg/dL (normal 0.2-1.2 mg/dL), and direct fraction was 0.2 mg/dL (normal 0.0-0.3 mg/dL). Serum amylase was 68 U/L (30-110 U/L) and lipase 34 U/L (10-60 U/L). The complete blood count was relevant for a white blood cell count of 7.2 × 10^3^/µL (4.0-11.0 × 10^3^/µL), hemoglobin of 12.9 g/dL (12.0-15.5 g/dL), hematocrit of 38.4% (36%-46%), mean corpuscular volume of 88 fL (80-100 fL), and platelets of 264 × 10^3^/µL (150-400 × 10^3^/µL). Creatinine was 0.8 mg/dL with normal serum albumin and C-reactive protein levels. The patient was admitted for further workup and pain control. The hematological, biochemical, and inflammatory parameters obtained on admission, during hospitalization, and at six-month follow-up are summarized in Table 1, and the metabolic risk factor profile before, at, and after presentation is presented in Table 2.

**Table 1:** Hematological, biochemical, and inflammatory parameters during hospitalization and at follow-up NP, not performed; WNL, within normal limits (result documented as normal in the clinical record; numerical value not recorded). WNL entries: Alanine aminotransferase, aspartate aminotransferase, gamma-glutamyl transferase, alkaline phosphatase, creatinine, serum albumin, and C-reactive protein were within their respective reference ranges at admission as documented in the clinical record; numerical values were not retrievable. Reference intervals are those in routine use at the reporting laboratory and are expressed in conventional units.

Parameter	Unit	Reference range	On admission	Repeat during admission	6-month follow-up
Hematology					
White blood cell count	×10³/µL	4.0-11.0	7.2	8.9	6.4
Neutrophils	%	40-75	NP	68	NP
Hemoglobin	g/dL	12.0-15.5	12.9	11.4	13.6
Hematocrit	%	36-46	38.4	34.6	40.8
Mean corpuscular volume	fL	80-100	88	86	90
Platelet count	×10³/µL	150-400	264	312	258
Liver panel					
Alanine aminotransferase	U/L	7-35	22	WNL	20
Aspartate aminotransferase	U/L	8-33	25	WNL	23
Gamma-glutamyl transferase	U/L	9-36	28	WNL	24
Alkaline phosphatase	U/L	35-104	76	WNL	71
Total bilirubin	mg/dL	0.2-1.2	0.7	NP	0.6
Direct bilirubin	mg/dL	0.0-0.3	0.2	NP	NP
Pancreatic enzymes					
Amylase	U/L	30-110	68	NP	NP
Lipase	U/L	10-60	34	NP	NP
Renal, nutritional and inflammatory					
Creatinine	mg/dL	0.5-1.1 (women)	0.8	WNL	NP
Serum albumin	g/dL	3.5-5.2	WNL	NP	NP
C-reactive protein	mg/L	<5	WNL	NP	NP
Markers of ischemia					
Serum lactate	mmol/L	0.5-2.2	NP	1.5	NP
Lactate dehydrogenase	U/L	140-280	NP	208	NP

**Table 2:** Metabolic risk factor profile before, at, and after presentation HbA1c, glycated hemoglobin A1c; HDL, high-density lipoprotein; LDL, low-density lipoprotein. ᵃThe reference range shown (HbA1c <5.7%) applies to the non-diabetic population. The guideline-recommended treatment target for most adults with type 2 diabetes mellitus is <7.0%. Lipid targets are those recommended for high cardiovascular-risk patients. All lipid and glucose values are expressed in conventional units (mg/dL and %, respectively).

Parameter	Unit	Reference/target value	1 year before presentation	On admission	6 months after revascularization
Total cholesterol	mg/dL	Desirable <200	268	360	172
LDL cholesterol	mg/dL	Optimal <100	179	230	88
HDL cholesterol	mg/dL	Desirable >50 (women)	38	32	46
Non-HDL cholesterol	mg/dL	Desirable <130	230	328	126
Triglycerides	mg/dL	Normal <150	247	347	148
Glycated hemoglobin (HbA1c)	%	Normal <5.7^a^	7.2	8.2	6.7

Upper GI endoscopy revealed extensive inflammatory and ulcerative changes in the stomach and duodenum. Gastric antrum and body presented severely edematous and erythematous mucosa with multiple scattered clean-based ulcers of different sizes and several scattered erosions (Figure 1A). There were multiple erosions on an erythematous edematous antrum. The proximal duodenum was erythematous with a large, clean-based ulcer and several smaller, scattered erosions from which biopsies were taken (Figure 1B).

**Figure 1:**
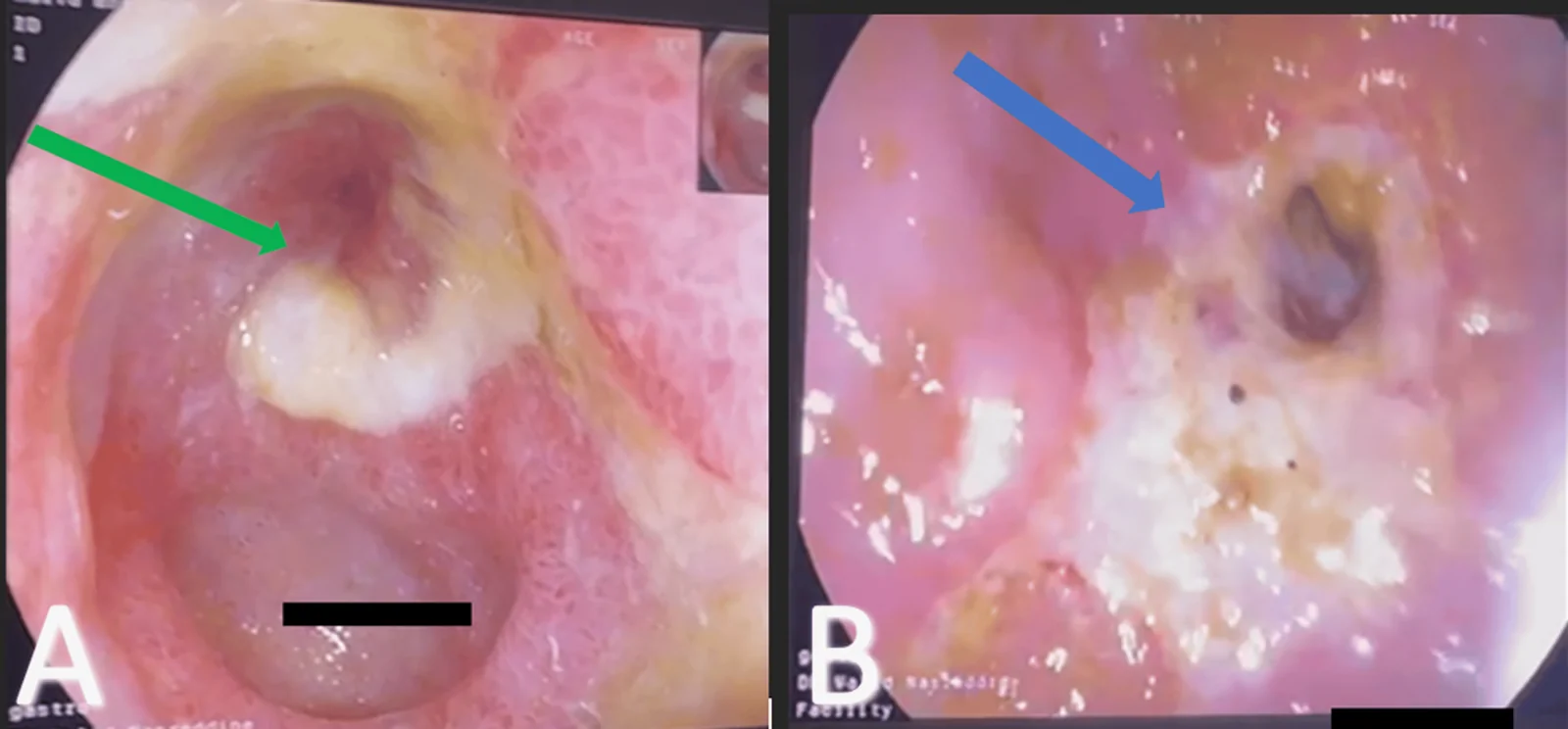
Upper gastrointestinal endoscopy findings (A) The green arrow indicates an erythematous first portion of the duodenum with a large, clean-based ulcer and several smaller, scattered erosions. (B) The blue arrow indicates a large, clean-based ulcer surrounding the antrum.

Moreover, erythematous mucosa was found in the second part of the duodenum. Endoscopic examination revealed severe erosive gastroduodenitis with multiple gastric ulcers and a large duodenal ulcer. The diffuse, continuous distribution of the lesions involving the fundus, body, antrum and duodenum was atypical for conventional peptic ulcer disease and, in association with the severity of the postprandial pain, progressive food aversion and marked weight loss, raised suspicion of ischemic etiology. The differential diagnosis was a malignant process. Duodenal tissue rapid urease testing was negative, and *H. pylori* testing by histology was also negative. Histopathology of the gastric and duodenal biopsies showed mucosal ulceration with granulation tissue (Figure 2A). Ulcerated duodenal mucosa replaced by fibro-leukocytic and necrotic material (Figure 2B).

**Figure 2:**
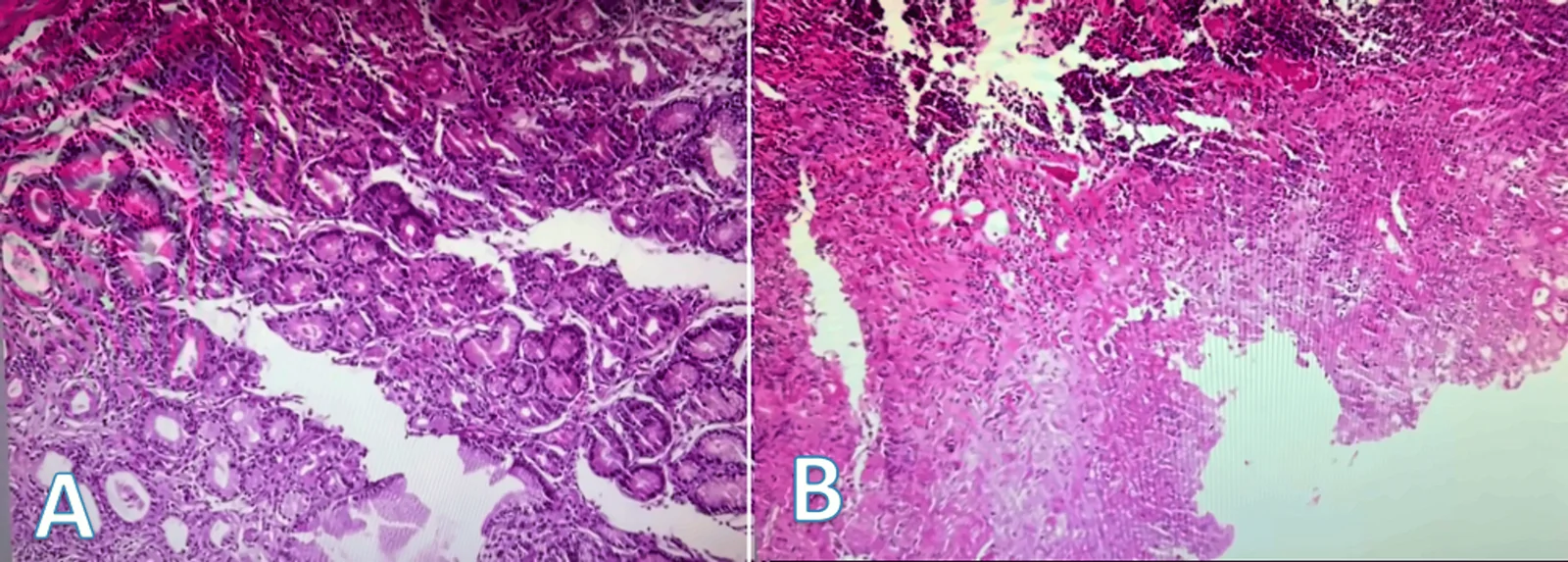
Histopathology of gastric and duodenal biopsies (A) Gastric antral mucosa demonstrating mild active gastritis with mucosal ulceration and underlying granulation tissue (hematoxylin and eosin, ×20). (B) Duodenal mucosa demonstrating surface ulceration, with the epithelium replaced by fibrinoleukocytic exudate and necrotic debris (hematoxylin and eosin, ×10).

There was no evidence of malignancy, dysplasia, viral inclusions or *H. pylori* organisms. The results of these ancillary and qualitative investigations, including *Helicobacter pylori* testing and the histopathological findings, are summarized in Table 3.

**Table 3:** Ancillary and qualitative investigations All biopsies were obtained during the index upper gastrointestinal endoscopy unless otherwise stated. Histopathological assessment was performed by a consultant gastrointestinal pathologist. Fecal occult blood test was performed using a guaiac-based method on a rectal examination specimen at the time of admission.

Test	Specimen/timing	Result
Fecal occult blood test	Rectal examination, admission	Negative
Rapid urease test	Duodenal biopsy, index endoscopy	Negative
*Helicobacter pylori* histology	Gastric and duodenal biopsies	Negative
Histopathology, gastric biopsy	Index endoscopy	Mucosal ulceration with granulation tissue; mild active antral gastritis with glandular activity; no malignancy or dysplasia
Histopathology, duodenal biopsy	Index endoscopy	Ulcerated mucosa replaced by fibro-leukocytic and necrotic material; no malignancy, dysplasia, or viral inclusions

Contrast-enhanced CTA of the abdomen demonstrated extensive atherosclerotic disease involving the major mesenteric vessels (Figures 3A, 3B). Figure 3A shows a sagittal maximum-intensity projection demonstrating near-complete stenosis of the celiac trunk (red arrow) and severe atherosclerotic stenosis at the origin of the superior mesenteric artery (green arrow), with marked calcification of the abdominal aorta. Figure 3B shows a sagittal soft-tissue window of the same region, demonstrating the stenotic origin of the celiac trunk (red arrow) and the proximal superior mesenteric artery.

**Figure 3:**
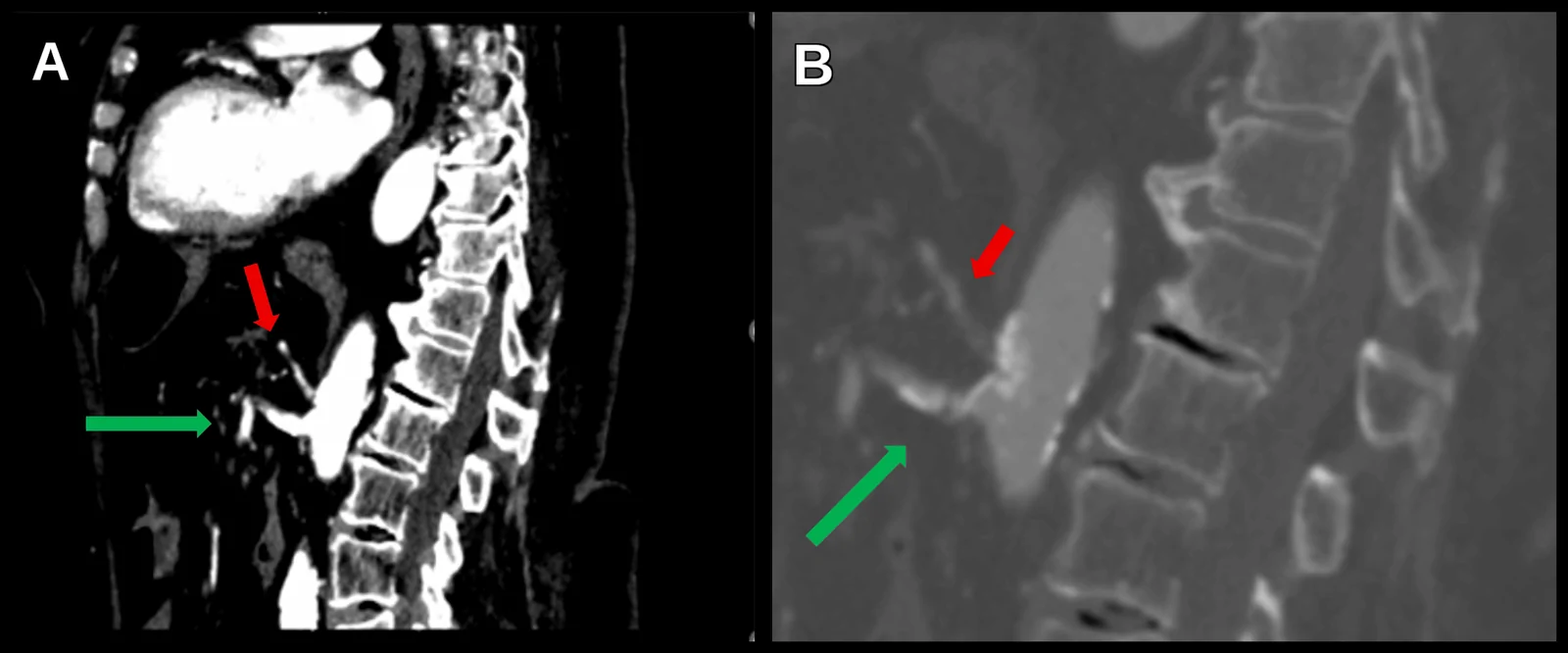
Contrast-enhanced computed tomography angiography (A) Sagittal maximum-intensity projection demonstrating near-complete stenosis of the celiac trunk (red arrow) and severe atherosclerotic stenosis at the origin of the superior mesenteric artery (green arrow), with marked calcification of the abdominal aorta. (B) Sagittal soft-tissue window of the same region showing the stenotic celiac trunk origin (red arrow) and the proximal superior mesenteric artery (green arrow).

The abdominal aorta was tortuous and markedly atherosclerotic, most severe at the origin of the celiac trunk, with a focal reduction in aortic caliber of approximately 60%. The celiac trunk was severely narrowed with near-complete stenosis over a segment of approximately 5 mm. The celiac trunk was reconstituted distally before giving off its major branches, but was markedly reduced in caliber. There was atherosclerotic calcification and marked narrowing of the common hepatic, left gastric, and splenic arteries. There was severe atherosclerotic disease at the origin and proximal segment of the SMA with approximately 60% narrowing at the origin and a 3-cm segment of severe stenosis including portions of near-complete luminal occlusion. The distal SMA was seen reconstituted with atheromatous calcification. The inferior mesenteric artery (IMA) was relatively well preserved in caliber with approximately 40% stenosis at its origin. There was good collateral circulation via the pancreaticoduodenal arcade and the arc of Riolan. There was no gross bowel-wall thickening or areas of mucosal hypo-enhancement to suggest acute bowel ischemia.

Repeat lab studies showed white blood cell count of 8.9 × 10^3^/µL with 68% neutrophils, hemoglobin of 11.4 g/dL, hematocrit of 34.6%, mean corpuscular volume of 86 fL, and platelets of 312 × 10 3/µL. Serum lactate was 1.5 mmol/L (normal range, 0.5-2.2 mmol/L), and lactate dehydrogenase was 208 U/L (normal range, 140-280 U/L); both values were within the normal ranges. Creatinine and liver enzymes were within normal limits. Repeat metabolic testing after a period of non-adherence to medications revealed markedly uncontrolled cardiovascular risk factors: total cholesterol 360 mg/dL, low-density lipoprotein (LDL) cholesterol 230 mg/dL, high-density lipoprotein (HDL) cholesterol 32 mg/dL, non-HDL cholesterol 328 mg/dL, triglycerides 347 mg/dL, and HbA1c of 8.2% (Tables 1, 2).

The case was reviewed in a multidisciplinary meeting with the gastroenterology, vascular and interventional radiology, and surgical teams. The diagnosis of chronic mesenteric ischemia with presentation of ischemic gastroduodenitis was made based on the typical clinical presentation, endoscopic findings, and evidence of severe multivessel mesenteric atherosclerotic disease. Initial management involved intravenous proton pump inhibitor therapy (40 mg twice daily), bowel rest with intravenous fluid, nutritional support, and analgesia. Secondary prevention consisted of reinstitution of atorvastatin 40 mg once daily (OD) with fenofibrate, initiation of antiplatelet therapy with clopidogrel 75 mg OD and optimization of glycemic control with structured counselling regarding medication adherence.

In view of the severity of her symptoms, amount of weight loss, inability to maintain adequate oral intake, and amount of stenotic disease within the mesenteric circulation, revascularization was felt to be necessary. The patient and family were counselled in detail about the endovascular intervention, the potential benefits, and the risks of the procedure. The decision to proceed with endovascular angioplasty and stenting of the SMA was made after a multidisciplinary evaluation and shared decision-making.

Recanalization of the SMA was technically challenging due to the near-occlusive stenosis and severe calcified atherosclerotic disease. There was a failure of several initial attempts to cross the obstruction, but the lesion was crossed, and angioplasty was successfully performed through right common femoral artery approach with ultrasound guidance. Balloon angioplasty was complicated by intraluminal thrombus and focal dissection of the proximal SMA, and repeat balloon dilatation was performed followed by placement of two overlapping balloon-mounted metallic stents (6 × 27 mm and 6 × 17 mm). Both stents were then redilated to 6 mm. Completion angiography showed no residual stenosis with brisk antegrade flow through the SMA branches (Figures 4A, 4B).

**Figure 4:**
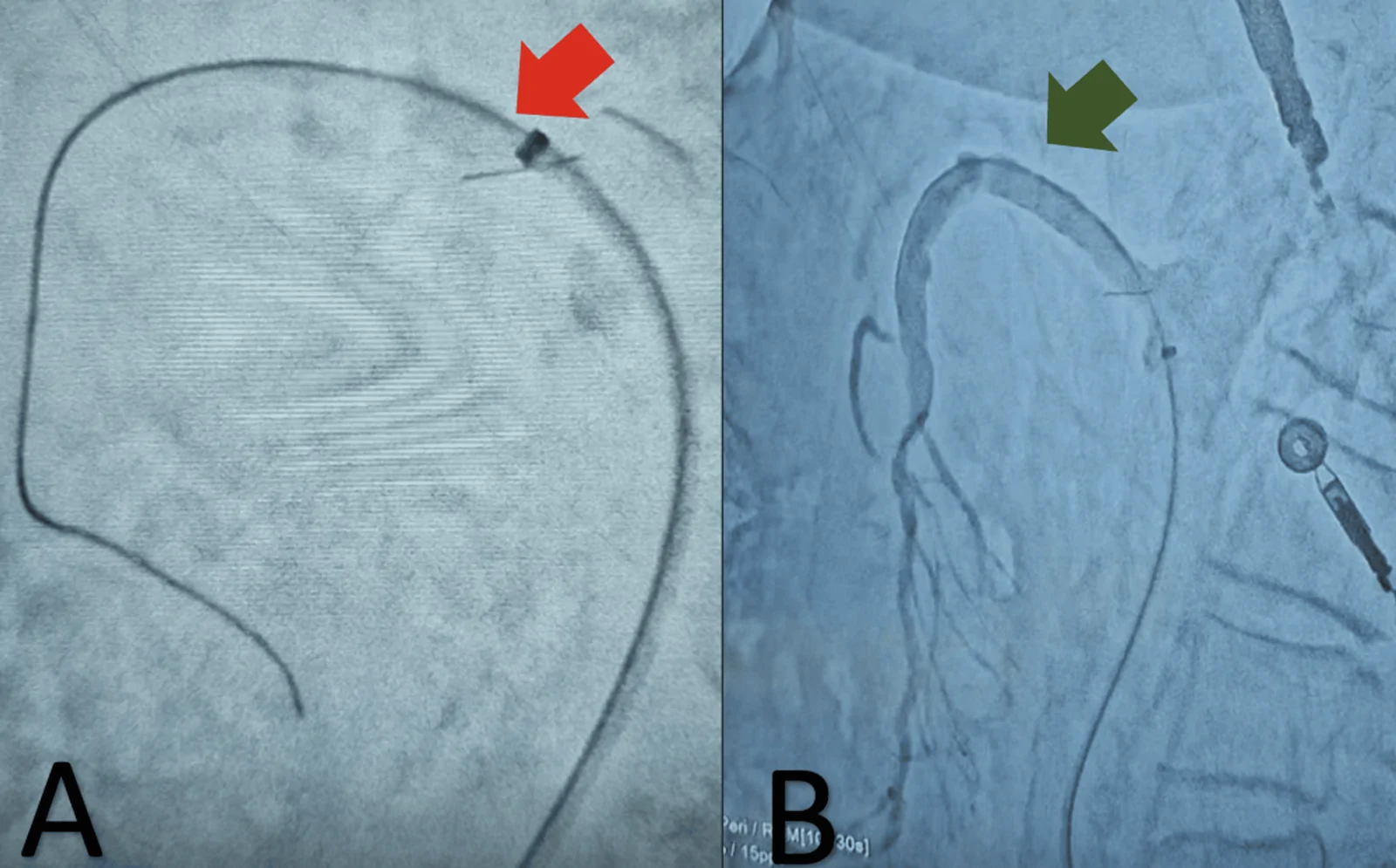
Digital subtraction angiography of the superior mesenteric artery (A) SMA angiography prior to stenting showing complete occlusion with absent contrast flow (red arrow). (B) SMA artery angiography after stenting demonstrating restored patency with antegrade contrast opacification (green arrow). SMA, superior mesenteric artery.

The procedure was well tolerated by the patient with no immediate peri-procedural complications. The total duration of hospitalization was 11 days. The patient was discharged on dual antiplatelet therapy, acetylsalicylic acid 100 mg once daily and clopidogrel 75 mg once daily, a strict low-fat diet, fenofibrate and atorvastatin once daily for aggressive secondary prevention of her severe, previously uncontrolled dyslipidemia. The patient had rapid resolution of her postprandial pain and advanced to a full, unrestricted oral diet within 30 days of the procedure. Repeat upper gastrointestinal endoscopy (EGD) performed four months after the stenting procedure and upon follow-up demonstrated complete mucosal healing of the previously identified ulcers involving the stomach and first portion of the duodenum (Figures 5A, 5B).

**Figure 5:**
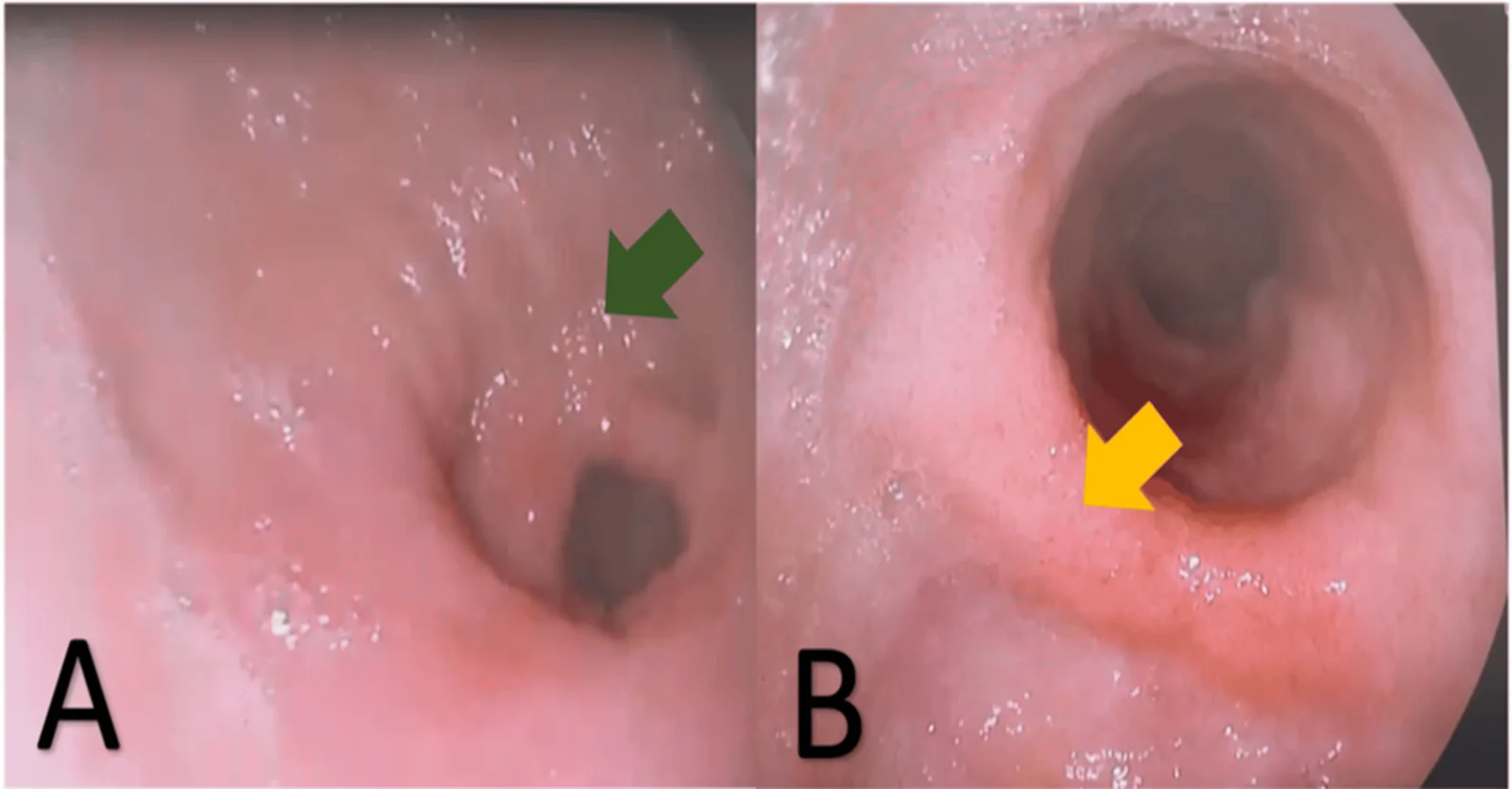
Follow-up upper gastrointestinal endoscopy (A) Complete healing of the previously described antral ulcer following SMA stenting (green arrow). (B) Complete healing of the previously described ulcer in the first part of the duodenum following SMA stenting (yellow arrow). SMA, superior mesenteric artery.

Then, she was switched to single agent therapy with acetylsalicylic acid 100 mg once daily after the second EGD, high-intensity statin therapy, fenofibrate, and intensified glycemic management. At six-month follow-up, she was asymptomatic and had gained 6 kg. She was also referred for follow-up CTA to assess stent patency, but this had not been performed at time of writing. At this visit, laboratory evaluation was repeated and was normal, with a normal complete blood count and liver enzyme panel. White blood cell count was 6.4 × 10³/µL (4.0-11.0 × 10³/µL), hemoglobin 13.6 g/dL (12.0-15.5 g/dL), hematocrit 40.8% (36-46%), mean corpuscular volume 90 fL (80-100 fL), and platelet count 258 × 10³/µL (150-400 × 10³/µL). Liver enzymes were normal (alanine aminotransferase 20 U/L (7-35 U/L), aspartate aminotransferase 23 U/L (8-33 U/L), gamma-glutamyl transferase 24 U/L (9-36 U/L in women), alkaline phosphatase 71 U/L (35-104 U/L), and total bilirubin 0.6 mg/dL (0.2-1.2 mg/dL)). These findings, together with her continued clinical improvement, supported continued mesenteric patency and were reassuring against recurrent ischemic injury or a delayed hepatobiliary complication of revascularization. Lipid profile repeated at this visit showed marked improvement from the markedly deranged values documented on admission, reflecting the combined effect of the low-fat diet, combination lipid-lowering therapy with atorvastatin and fenofibrate and restored medication adherence after discharge. Total cholesterol was reduced to 172 mg/dL (desirable <200 mg/dL) from 360 mg/dL on admission, LDL cholesterol to 88 mg/dL (optimal <100 mg/dL) from 230 mg/dL, and triglycerides to 148 mg/dL (normal <150 mg/dL) from 347 mg/dL. HDL cholesterol was 46 mg/dL (desirable >50 mg/dL in women) after 32 mg/dL, and non-HDL cholesterol was 126 mg/dL (desirable <130 mg/dL) after 328 mg/dL. Similarly, HbA1c had improved from 8.2% on admission to 6.7%. This important metabolic improvement highlights the value of combining mechanical revascularization with aggressive and sustained secondary prevention in patients with atherosclerotic disease, partly driven by longstanding non-adherence.

## Discussion

Gastric ischemia is a rare clinical entity because of the extensive collateral arterial network of the stomach supplied by the left and right gastric, gastroepiploic, and short gastric arteries. Consequently, clinically important ischemia is usually observed only with multiple vascular territories or severe systemic hypoperfusion. Progressive atherosclerotic disease of the celiac artery and SMA is the most common cause of CMI and the leading cause of ischemic gastritis in elderly patients with cardiovascular risk factors [1-3]. The classic triad of CMI includes postprandial abdominal pain, food avoidance, and progressive weight loss, known as “abdominal angina”. However, gastric ischemia can also manifest with nonspecific gastrointestinal symptoms such as nausea, vomiting, upper gastrointestinal bleeding, or refractory gastric ulceration. These presentations are often delayed in diagnosis as they can be mistaken for more common conditions such as peptic ulcer disease and gastric malignancy [1,7]. In our patient, the severe postprandial epigastric pain, inability to tolerate oral intake and marked gastric and duodenal ulceration initially suggested a diagnosis of peptic ulcer disease. Histopathology was negative for malignancy. *H. pylori* testing was negative, and there was no history of nonsteroidal anti-inflammatory drug exposure. CTA subsequently demonstrated severe occlusive disease of the mesenteric arteries confirming the diagnosis of chronic ischemic gastroduodenitis. Note the absence of the two principal causes of gastroduodenal ulceration. In an *H. pylori*-negative patient without nonsteroidal anti-inflammatory drug (NSAID) exposure, diffuse, continuous ulceration of the fundus, body, antrum, and duodenum is difficult to explain by conventional peptic ulcer disease. This constellation should raise consideration of an ischemic etiology in a patient with vascular risk factors. Another point of practical importance is that our patient had normal serum lactate and lactate dehydrogenase levels despite extensive mucosal injury. In CMI, hypoperfusion is transient, demand-driven, and, in the absence of transmural infarction, there is little spillover of anaerobic metabolites into the systemic circulation. Normal lactate therefore does not exclude mesenteric ischemia; lactate levels may rise late and are predominantly elevated in acute mesenteric ischemia with established necrosis. This is illustrated in the case reported by Lundsmith et al. where lactate was elevated only once the patient had progressed to gastric perforation [7]. Normal lactate level is an established predictor of diagnostic delay. CTA is the investigation of choice in suspected cases of mesenteric ischemia as it can accurately localize and determine the degree of arterial stenosis along with assessment of bowel and gastric perfusion [2,6]. Endoscopy remains an important diagnostic tool, as it allows for direct visualization of mucosal injury and biopsy to exclude malignant, infectious, and medication-related causes of ulceration. Nevertheless, endoscopic findings are non-specific and must always be correlated with the clinical presentation and vascular imaging [1].

CMI management has been affected by two recent updates. In 2021, the Society for Vascular Surgery (SVS) published clinical practice guidelines recommending an endovascular-first approach for most patients with atherosclerotic CMI, reserving open repair for younger, lower-risk patients with anatomy unfavorable for stenting, and for those with recurrent symptoms after a failed endovascular attempt [6,8]. This was confirmed and extended by the European Society for Vascular Surgery (ESVS) 2025 clinical practice guidelines on the management of diseases of the mesenteric arteries and veins and the renal arteries, replacing the 2017 ESVS guidelines, and provided 102 graded recommendations on chronic and acute mesenteric ischemia, median arcuate ligament syndrome and related vascular pathology [9-11]. The revised ESVS document maintains the preference for endovascular revascularization as first-line therapy for atherosclerotic CMI, broadens this recommendation to symptomatic in-stent re-stenosis following either endovascular or open repair, and introduces the issue of surveillance imaging (duplex ultrasound as first-line surveillance with CTA as second-line imaging when re-stenosis is suspected) [12,13]. Also complementary standards from the Cardiovascular and Interventional Radiological Society of Europe (CIRSE), published in 2025, prefer endovascular therapy as the primary treatment for arterial mesenteric ischemia, due to its minimally invasive nature and lower in-hospital mortality, while open surgery is reserved for cases with established or suspected bowel necrosis [14].

Open surgical revascularization and endovascular therapy are not seen as competing in the comparative literature, but rather as tools for different clinical situations. Registry-based and meta-analytic data have consistently shown that endovascular revascularization is associated with lower perioperative morbidity and mortality, shorter hospital stay and faster recovery of oral intake as compared with open bypass or endarterectomy, which is of particular importance in patients that are elderly and cachectic, like ours, and who poorly tolerate major abdominal surgery [4,6,15]. However, this short-term safety benefit comes at the cost of durability. Pooled analyzes and single-center comparative series report substantively higher long-term primary patency and freedom from recurrent symptoms with open revascularization, at the cost of a two- to three-fold higher rate of perioperative complications [15,16]. In a recent value analysis using longitudinal claims data, endovascular repair continued to provide higher five-year value per healthcare dollar than open surgery for the majority of patients, after adjustment for perioperative risk, reintervention, and life expectancy, supporting the role of endovascular repair as the default initial strategy, regardless of the fact that open surgery is more durable if successfully completed [17]. In practice, the decision is based on anatomy, physiologic reserve, and center expertise rather than diagnosis alone. In general, we prefer endovascular treatment in a disease limited to a short, focal segment that can be treated with angioplasty and stenting, as in the SMA lesion of our patient, and in patients with a comorbidity burden or nutritional state that makes them poor candidates for laparotomy [6,12]. Open revascularization remains indicated for long occlusive segments not suitable for stenting, for young or low-risk patients in whom durable long-term patency is desired, for failed or technically impossible endovascular recanalization, and for patients requiring concomitant laparotomy to evaluate or resect nonviable bowel [6,15,18]. Most centers including ours have adopted a hybrid or "endovascular-first" approach where percutaneous revascularization is attempted first and open surgery is reserved for technical failure or recurrent stenosis. This approach is explicitly supported for recurrent disease in the 2025 ESVS guidelines . Thus, interventional radiology and vascular surgery are best considered complementary components of a single revascularization pathway, rather than alternative competing therapies, and management decisions are best made collaboratively through the multidisciplinary discussion demonstrated in this case. In the present case, an endovascular-first approach was chosen in view of the patient’s age, cachexia and comorbidity burden, despite near-complete celiac artery occlusion and long technically challenging SMA stenosis. Successful angioplasty and stent placement allowed us to restore mesenteric perfusion. The patient rapidly became asymptomatic, was able to resume oral intake, and the extensive gastroduodenal ulceration completely healed on endoscopy, avoiding the need for open surgery (Table 4). Comparison of the current case with previously reported cases of gastric ischemia due to mesenteric arterial occlusive disease. There are some observations. First, affected patients are generally elderly and have a heavy burden of atherosclerotic risk factors, although ischemic gastropathy may also be precipitated in the setting of systemic hypoperfusion superimposed on fixed stenosis [9]. Second, the diagnostic approach is identical in all reports: endoscopy identifies the mucosal injury and excludes other causes, and cross-sectional or Doppler-based vascular imaging determines the etiology [3,10,11]. Third and most importantly, outcome appears to be largely dependent on timing of revascularization. All cases diagnosed and revascularized before transmural injury has occurred have had symptomatic relief and mucosal healing [3,9-11], while delayed recognition has resulted in progression to perforation and emergency laparotomy as in the case reported by Lundsmith et al., where attempted cannulation of the occluded SMA was unsuccessful and endovascular options had been lost [7]. Ischemic gastritis secondary to mesenteric arterial occlusive disease has been described in several case reports, but complete endoscopic resolution after isolated SMA revascularization has rarely been reported. Our case lends credence to the concept that restoration of mesenteric blood flow can reverse ischemic gastric injury even in the setting of severe celiac artery compromise, emphasizing the significance of collateral circulation, predominantly via the pancreaticoduodenal arcade, once SMA flow has been restored [3,8]. This case illustrates the importance of a high index of suspicion for gastric ischemia in the elderly with postprandial abdominal pain, weight loss and refractory gastric or duodenal ulcers in the setting of significant atherosclerotic disease. Early diagnosis, multidisciplinary approach and early endovascular intervention are essential to prevent progression to gastric necrosis, perforation and other life-threatening complications.

**Table 4:** Reported cases of gastric ischemia secondary to mesenteric arterial occlusive disease: presentation, diagnostic modality, treatment, and outcome CKD, chronic kidney disease; CT, computed tomography; CTA, computed tomography angiography; DAPT, dual antiplatelet therapy; EGD, esophagogastroduodenoscopy; IMA, inferior mesenteric artery; LDH, lactate dehydrogenase; MR, magnetic resonance; PPI, proton pump inhibitor; SMA, superior mesenteric artery; T2DM, type 2 diabetes mellitus.

Author (year)	Age/Sex	Presentation	Vascular lesions	Diagnostic tools	Treatment	Outcome
Lundsmith et al. (2016) [7]	69/M	3 months of diffuse abdominal pain worse with meals, weight loss, dysphagia	Celiac trunk and SMA occlusion; patent IMA	EGD; CT; CTA; mesenteric angiography	SMA cannulation unsuccessful; emergency laparotomy with repair of gastric perforation	Progression to gastric perforation; lactic acidosis (peak 7 mmol/L), intubation; poor outcome
Anucha et al. (2019) [9]	76/F	Upper gastrointestinal bleeding following sepsis and hypotension; hypertension, hyperlipidemia, dialysis-dependent CKD	Celiac artery stenosis	EGD; angiography	Interventional radiology-guided celiac artery stenting	Resolution of bleeding; ischemic gastropathy successfully treated
Yamamoto and Hashimoto (2023) [10]	82/M	Anorexia, refractory gastric ulcers unresponsive to high-dose PPI; dialysis-dependent CKD	Celiac artery stenosis (peak systolic velocity 270 cm/s)	EGD (“gastric double-stripe sign”); Doppler ultrasound; MR angiography	Endovascular revascularization of the celiac artery	Symptomatic relief with ulcer resolution; uneventful course; well at one year
Ribas et al. (2023) [3]	71/F	Severe ischemic gastritis with ulceration and upper gastrointestinal bleeding	Celiac trunk and IMA occlusion; critical SMA stenosis	EGD; CT angiography	SMA angioplasty with covered balloon-expandable stent (left brachial access); PPI, statin, cilostazol, then DAPT	Discharged on postoperative day 2; ulcers healed on EGD at 30 days; asymptomatic with 10 kg weight gain and patent stent at 6 months
Abdoh et al. (2023) [11]	55/M	6 months of epigastric pain worse after fatty meals, relieved by fasting, with weight loss; heavy smoker	SMA and IMA stenosis	Gastroscopy (*Helicobacter pylori*-negative antral ulcers); CT angiography	Endovascular intervention	Technically successful with clinical improvement
Present case (2026)	77/F	1 month of severe postprandial epigastric pain, food fear, intolerance of solids, 10 kg weight loss; uncontrolled T2DM and dyslipidemia	Near-complete celiac trunk occlusion; 3-cm severe SMA stenosis with near-occlusive segments; 40% IMA ostial stenosis	EGD with biopsy (*H. pylori*-negative, no malignancy); CT angiography; normal lactate and LDH	SMA angioplasty with stenting; PPI, statin, antiplatelet therapy, glycemic optimization	Rapid symptom resolution with resumption of oral intake; complete endoscopic healing of gastric and duodenal ulceration

## Conclusions

Chronic ischemic gastroduodenitis is an uncommon but important manifestation of chronic mesenteric ischemia and should be considered in elderly patients presenting with postprandial abdominal pain, weight loss and gastric or duodenal ulceration refractory to treatment, especially in the setting of significant atherosclerotic disease. Finally, a normal serum lactate should not be considered sufficient to exclude the diagnosis, and the finding of diffuse ulceration in the absence of *H. pylori* infection and NSAID use is a particularly important diagnostic clue. Early diagnosis by computed tomography angiography and prompt endovascular revascularization may result in rapid symptom resolution and complete mucosal healing. The case emphasizes the significance of keeping a high index of suspicion and a multidisciplinary approach to improve patient outcomes.
